# DermaDashboard: Bridging the Gap Between FHIR Standards and Clinical Usability

**DOI:** 10.2196/73691

**Published:** 2025-10-22

**Authors:** Katarzyna Borys, Eva Maria Hartmann, Ahmad Idrissi-Yaghir, Elisabeth Livingstone, Georg Lodde, Cynthia Sabrina Schmidt, Philipp Winnekens, Christoph M Friedrich, René Hosch, Felix Nensa

**Affiliations:** 1 Institute for Artificial Intelligence in Medicine, Essen University Hospital, Essen, Germany,; 2 Institute of Diagnostic and Interventional Radiology and Neuroradiology, Essen University Hospital, Hufelandstraße 55, Essen, 45147, Germany, 49 201-723-77818; 3 Department of Computer Science, University of Applied Sciences and Arts Dortmund, Dortmund, Germany; 4 Department of Dermatology, Venereology, and Allergology, Essen University Hospital, Essen, Germany; 5 Institute of Transfusion Medicine, Essen University Hospital, Essen, Germany; 6 Institute for Medical Informatics, Biometry, and Epidemiology (IMIBE), Essen University Hospital, Essen, Germany

**Keywords:** melanoma, dashboard, FHIR, data acquisition, interoperability, analytics, Fast Healthcare Interoperability Resources

## Abstract

Over the past decade, Fast Healthcare Interoperability Resources (FHIR) have become increasingly relevant in health care data standardization. However, the complex structure of FHIR makes cohort analytics with many-to-many relations extremely time-consuming, and, impossible in many cases. To support exploratory cohort building and data visualization in oncology, especially for nontechnical users, we developed the DermaDashboard, an interactive dashboard built on top of a relational FHIR-compliant PostgreSQL database. Relevant oncology data was preaggregated with a materialized view, and the subsequent visualization layer was implemented using an open-source visualization tool, enabling clinicians to filter and analyze data without requiring familiarity with FHIR or SQL. The database encompassed data from 3949 patients with melanoma and included 82,783 health records. Core FHIR resources were Patient, DiagnosticReport, and QuestionnaireResponse, with 54 mapped attributes spanning demographics, stagings, mutations, and treatments. The resulting dashboard allowed filtering across 29 variables to construct subcohorts and generate aggregation analyses. This implementation shows how open interoperability data standards, such as FHIR, can be used in the development of modular, user-friendly clinical dashboards for cohort analysis, and the architecture demonstrates a feasible path toward democratizing access to structured health care data.

## Introduction

In recent years, health care systems have rapidly embraced digitalization to enhance patient care, streamline clinical workflows, and improve interoperability of health information [1-4]. At the heart of this transformation is the widespread adoption of electronic health records (EHRs) [5], which, on a broader scale, support the secondary use of data beyond clinical aspects, such as quality control, safety measurements, payments, certification, and research [6,7]. However, hospitals employ diverse IT systems that store data in incompatible formats, thereby hindering cross-system analytics. The demand for interoperability among health care systems has thus become increasingly urgent. Among the technical standards driving this shift, the HL7 Fast Healthcare Interoperability Resources (FHIR) standard [8] has emerged as a cornerstone for the structured, machine-readable exchange of health care data [9,10]. By relying on modern web technologies, such as RESTful APIs (Representational State Transfer Application Programming Interfaces), FHIR enables scalable, modular data interoperability across diverse health care systems [11-13].

The application of FHIR in analytics and research, however, remains limited due to the standard’s nested data structure. FHIR is a deeply nested graph, which is why analytics with many-to-many relationships require countless chained queries over the RESTful API. Because of this, analytics become slow and often impractical for advanced insights.

Additionally, most hospital information systems remain limited in scope, as they are primarily designed for viewing individual patient records. Consequently, they often lack functionalities for population-level analytics or cohort-based data exploration. Furthermore, raw EHR data is usually heterogeneous, incomplete, and multimodal [14], making it difficult to query directly, especially for nontechnical users. These structural constraints hinder the ability to assemble complex patient cohorts and quickly extract statistics, capabilities that are essential for data-driven care and clinical research.

As health care institutions increasingly adopt FHIR to standardize data, there is a growing need for tools that enable accessible, exploratory analysis of FHIR-based datasets without requiring deep technical knowledge. In particular, there is an unmet need for lightweight, modular tools that can bridge the gap between the complex FHIR data standard and intuitive clinical interfaces used by health care professionals.

As a mitigating solution, we developed DermaDashboard, a proof-of-concept for a FHIR-based dashboard designed to facilitate exploratory cohort analysis for oncology research across multiple IT systems. Built using a commercial relational FHIR model and an open-source visualization tool, this dashboard enables structured cohort filtering, data summarization, and visual exploration. Demonstrated using patients with melanoma, the architecture is modular and generalizable to other diseases. This viewpoint provides a technical overview of the development pipeline, highlighting how FHIR-based data infrastructures can support the development of accessible, standards-driven dashboards in clinical environments.

## FHIR in Analytics: Challenges and Solutions

FHIR represents health care data as modular resources, each capturing specific clinical information which are linked via unique identifiers [15]. While FHIR supports interoperability through RESTful APIs, assembling complex, multicriteria patient cohorts and performing cohort-level analyses can be challenging with conventional REST queries due to the hierarchical and deeply nested structure of the data [16,17].

To address these practical challenges, our institution uses the relational FHIR engine provided by Firemetrics [18], which stores FHIR resources in a relational PostgreSQL database without requiring custom mapping, maintaining full compliance with the FHIR standard. This relational representation allows preprocessing via SQL scripts to validate, clean, and normalize real-world clinical data, while also enabling efficient cohort-level queries and flexible integration with analytical dashboards. By combining the interoperability of FHIR with the performance and versatility of relational queries, the system supports facilitated data workflows.

Importantly, although our implementation uses Firemetrics [18] for relational FHIR storage, the architectural approach is modular and not restricted to a single platform. It can be adapted to other systems that provide comparable functionality. For example, *fhirbase* [19] provides a relational persistence layer for FHIR resources, while SQL-on-FHIR [20] specifies a standard for expressing queries against FHIR data without prescribing a concrete implementation. In addition, FHIR servers such as Blaze [21] offer an alternative infrastructure that can be extended with complementary components to support cohort-level querying and integration with analytical tools, thereby underscoring the flexibility and generalizability of the proposed framework.

## A Relational, Modular Approach to Clinical Data Integration

The first step of the pipeline is the assembly of a materialized view within the relational database. This materialized view serves as a precomputed layer that aggregates only the clinically relevant data from FHIR resources, specifically focusing on patients diagnosed with malignant melanoma and treated at the Department of Dermatology in the University Hospital Essen. Filtering needed data upfront reduces computational overhead and query times during downstream analyses. The construction of this view draws data from three primary FHIR resources:

1. Patient: providing demographic and identification data,
2. DiagnosticReport: containing structured clinical documentation, and
3. QuestionnaireResponse: holding detailed tumor documentation information in a structured but deeply nested format

Melanoma cases were identified by filtering the QuestionnaireResponse resource using standardized International Classification of Diseases (ICD)-10 German Modification [22] codes (C43*). The initiation script of the materialized view (see code in Multimedia Appendix 1) gathers each patient’s demographic, diagnostic, histopathological, therapeutic, clinical, procedural, genetic, and consent information. The whole palette of required information for patients with melanoma was informed by interdisciplinary collaboration with dermatologists and prior research at our institution, which identified key data requirements for management of patients with melanoma via a dashboard, as shown by Hartmann et al [23].

Due to the heterogeneous and semistructured nature of real-world hospital data, additional transformation steps were necessary to harmonize the data. These included standardizing inconsistent terminology, cleaning malformed or annotated values, parsing nonstandardized or missing datetime entries, and de-duplicating redundant records. These preprocessing steps were implemented during the initialization of the materialized view using a combination of SQL functions (eg, split_part, CASE) and JSONPath queries [24], which enabled the extraction and modification of structured data from nested JSONB fields.

## Dashboard Design and Visualization

Using the FHIR resources *Patient*, *DiagnosticReport*, and *QuestionnaireResponse*, we assembled a dataset of 3949 patients with melanoma (47% female), comprising 82,783 records as of February 12, 2025. Multiple entries per patient document recurring clinical events, such as staging, therapies, surgeries, and side effects, capturing disease progression over time.

The visualization layer of *DermaDashboard* was implemented in Grafana (v9.5.3; Grafana Labs)[25], which directly accesses the underlying database. As these queries deliver data in a usable format, no postprocessing is needed, enabling highly flexible ad-hoc analyses. Institutions with an existing FHIR server can replicate this open-source setup.

**Figure 1.**
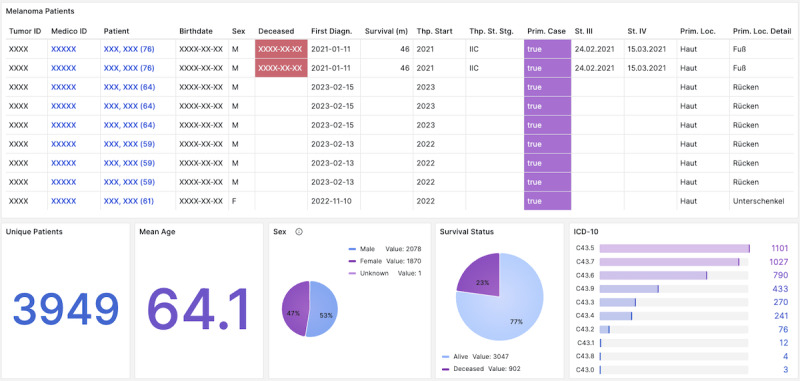
Outline of the Dashboard table panel: the main element of the Dashboard is the table panel, which presents all relevant patient information in tabular form. The presented outline displays deidentified patients, with a subset of columns focusing on demographics, therapy details, histological specifications, and mutation status.

**Figure 2.**
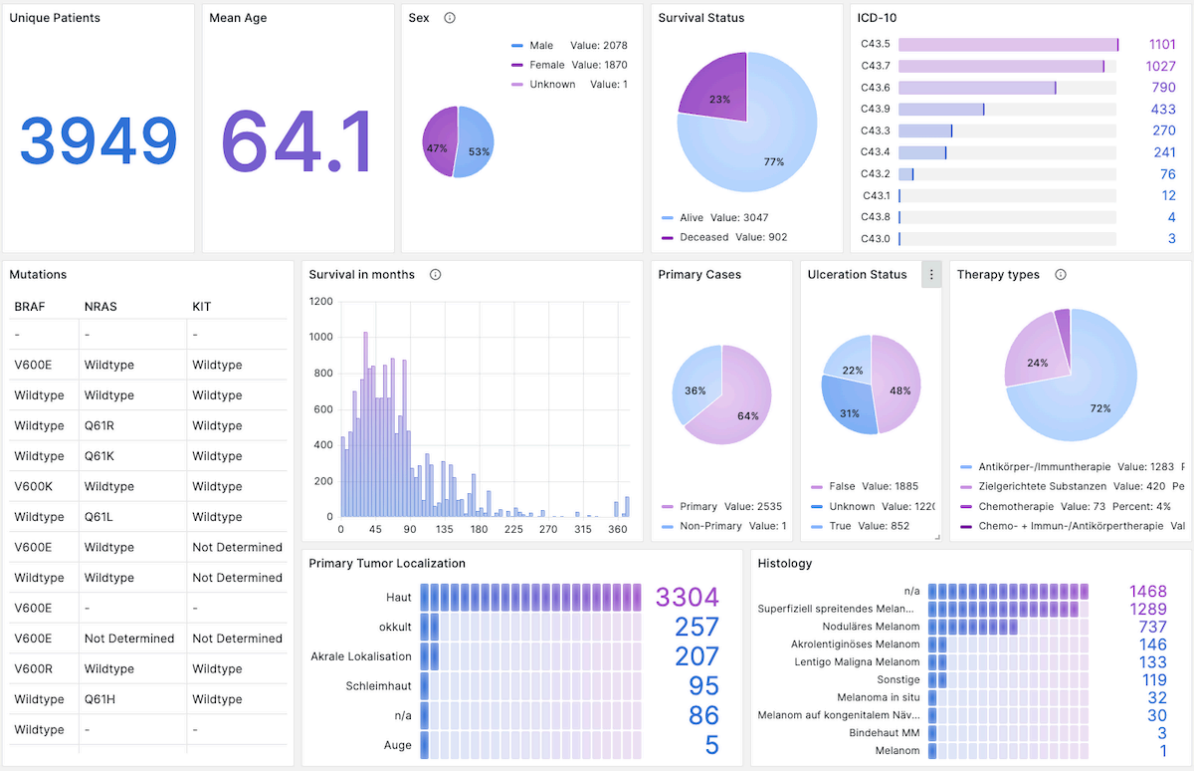
Outline of the dashboard’s subpanels: subsequent panels provide analytic information about the defined cohort by presenting count metrics for tumor locations, histological subtypes, and genetic mutations. Furthermore, the distributions of survival in months, primary cases, ulceration statuses, and procedure types are displayed.

The central element of the dashboard is a configurable table (Figure 1) that integrates 54 data columns spanning demographics, diagnostics, histopathology, therapies, procedures, genetic information, and consent (see query in Multimedia Appendix 2). Subpanels (Figures2  3) provide instant cohort statistics (eg, age, sex, survival, ICD-10 codes, tumor subtypes, genetic mutations, procedures, therapy outcomes) and allow filtering by 29 clinical variables (see full description in Multimedia Appendix 3). All panels update dynamically when filters are applied.

**Figure 3.**
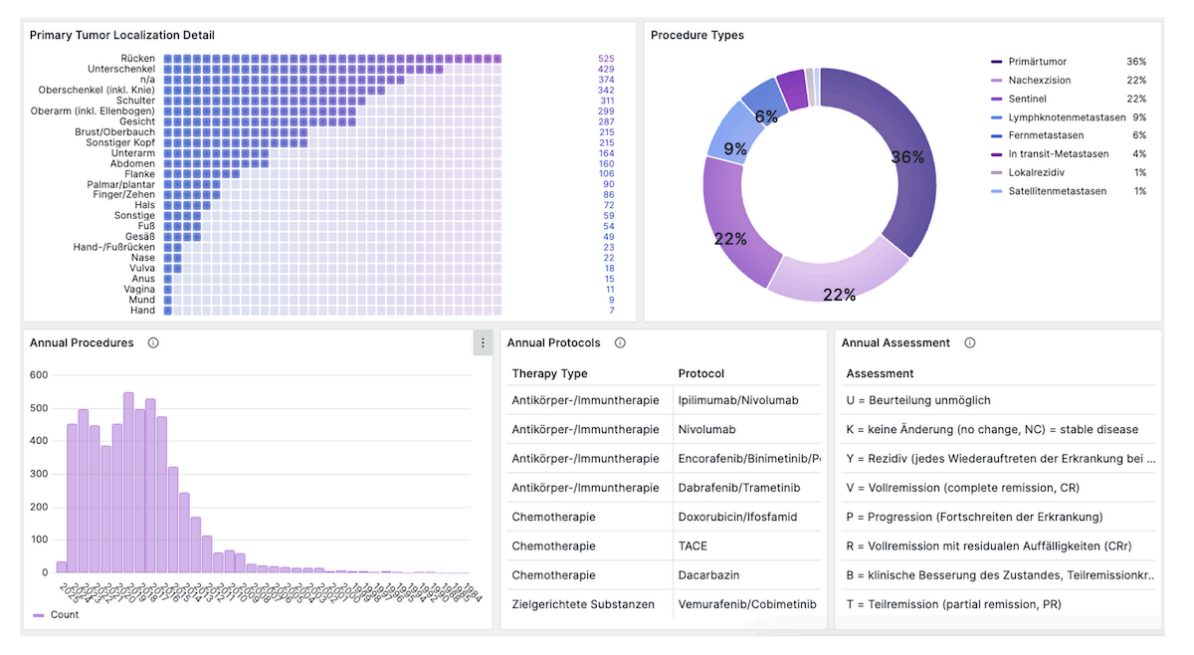
Annual analyses in the dashboard: specific panels focus on the annual analysis of performed surgical procedures and administered therapy protocols.

To facilitate analyses, Grafana supports a CSV export of defined cohorts. A custom deidentification variable removes names, dates, and identifiers before export. Finally, the dashboard is deployed securely in a locally hosted environment, ensuring compliance with institutional and legal data-protection standards.

## Barriers to Broader Adoption

While the modular dashboard pipeline we describe demonstrates the feasibility of FHIR-based analytics, broader adoption may be constrained by several practical factors. The complexity of FHIR’s hierarchical and nested structure remains a challenge to widespread use, even as relational implementations offer promising alternatives. Our system addresses this by using a relational FHIR model and SQL-based transformations, but this approach requires domain-specific expertise that may not be readily available at all institutions.

Technical development and maintenance currently demand substantial internal knowledge of health care data. This reliance on specialized skills could limit replication, especially in smaller or less technically equipped settings. In its present form, the dashboard also focuses exclusively on structured data; its utility for unstructured data or multimodal integration remains unexplored.

Further, DermaDashboard has not yet undergone a formal clinical or usability evaluation. Its practical impact on workflows, decision-making, or outcomes has yet to be validated. Similarly, no comparative assessment has been conducted against other FHIR-based analytics tools or commercial dashboard platforms. These open questions merit exploration in future work, ideally using unified datasets and evaluation frameworks. Finally, the current system’s focus on melanoma constrains immediate generalizability, though its architecture is designed to support broader use cases with minimal reconfiguration.

## Conclusion

The implementation of DermaDashboard presents a modular proof-of-concept for FHIR-based data analysis in a real-world clinical setting. Focused on melanoma, this pipeline addresses the challenge of translating complex health care data into a user-friendly format for downstream analysis and cohort acquisition, specifically for nontechnical users such as health care professionals. By combining diverse FHIR resources in a relational database and leveraging open-source visualization tools, the system enables accessible, reproducible cohort analysis tailored to institutional needs.

DermaDashboard showcases the practical utility of the FHIR standard by demonstrating how standardized, interoperable health care data can support user-friendly clinical tools. Its customizable filters, cohort-specific statistics, and CSV export functionality facilitate broad and flexible data access, especially to nontechnical users. The pipeline can be adapted to other disease domains through extended ICD code filtering and adjusted visualizations. While our implementation uses Firemetrics [18] for relational FHIR storage, the underlying approach could be applied to other relational FHIR platforms or tools, such as SQL-on-FHIR [20], fhirbase [19], Blaze [21], Grafana [25], Metabase [26], or Redash [27], enabling replication across institutions with varying technical environments.

While FHIR adoption is expanding, its use often remains confined to narrow research applications. In oncological practice, where multimodal data is frequently siloed, dashboards like DermaDashboard could enhance informed decision-making and identify data quality issues.

By offering a flexible, standards-based framework, DermaDashboard contributes to operationalizing FHIR for routine care and highlights a scalable path forward for interoperable, clinician-accessible analytics in health care.

## Supplementary material

10.2196/73691 Multimedia Appendix 1 SQL query used to initialize the materialized view for FHIR-based melanoma cohort preaggregation.

10.2196/73691 Multimedia Appendix 2 SQL query constructed within Grafana to retrieve data from the FHIR-based materialized view.

10.2196/73691 Multimedia Appendix 3 Overview of Grafana table panel column names, descriptions, and defined filtering variables.

## Abbreviations

API: Application Programming Interfaces
EHR: electronic health records
FHIR: Fast Healthcare Interoperability Resources
