# Supplementary material for: DermaDashboard: Bridging the Gap Between FHIR Standards and Clinical Usability
Source: JMIR Cancer. 2025 Oct 22;11:e73691. doi: 10.2196/73691 (PMC12543034; doi:10.2196/73691)
Supplement: Multimedia Appendix 3 [file cancer-v11-e73691-s003.docx]

# Table S1: Overview of all dashboard table columns

|  | **Column Name** | **Data Type** | **Description** |
| --- | --- | --- | --- |
| 1 | Tumor ID | String | Unique identifier for each tumor case. |
| 2 | Medico ID | String | Unique hospital information system ID. |
| 3 | Patient | String (Integer) | Displays patient name and age. |
| 4 | Birthdate | Date | Date of birth of the patient |
| 5 | Sex | String | Biological sex of the patient. |
| 6 | Deceased | Date | Date of death (if applicable). |
| 7 | First Diagn. | Date | Date of first melanoma diagnosis |
| 8 | Survival (m) | Integer | Total survival in months from initial diagnosis to death of any cause. |
| 9 | Thp. Start | Date | Date when therapy started at the institution. |
| 10 | Thp. St. Stg. | String | Cancer stage at the start of therapy. |
| 11 | Prim. Case | Boolean | Indicates if this is the primary tumor case. |
| 12 | St. III | Date | Date when stage III diagnosis was made (if applicable). |
| 13 | St. IV | Date | Date when stage IV diagnosis was made (if applicable). |
| 14 | Prim. Loc. | String | General primary melanoma location. |
| 15 | Prim. Loc. Detail | String | Specific anatomical site of the primary melanoma. |
| 16 | Histology | String | Histological classification of the melanoma. |
| 17 | ICD-10 | String | ICD-10 classification code for melanoma. |
| 18 | Ulceration | Boolean | Indicates if ulceration is present in the tumor. |
| 19 | BRAF | String | BRAF mutation status of the tumor. |
| 20 | NRAS | String | NRAS mutation status of the tumor. |
| 21 | KIT | String | KIT mutation status of the tumor. |
| 22 | Staging(c) Date | Date | Date of clinical staging. |
| 23 | Staging(c) Finding | Sring | Specific diagnosis of clinical finding. |
| 24 | T(c) | String | Clinical tumor classification (TNM: Tumor). |
| 25 | N(c) | String | Clinical nodal classification (TNM: Nodes). |
| 26 | M(c) | String | Clinical metastasis classification (TNM: Metastases). |
| 27 | Stage(c) | String | Overall clinical staging of the tumor. |
| 28 | At First Diagn. | Boolean | Indicates if the stage was assessed at first diagnosis. |
| 29 | Prcd. Date | Date | Date of surgical procedure. |
| 30 | Prcd. Type | String | Type of surgical procedure performed. |
| 31 | LAD | Boolean | Indicates if a lymphadenectomy was performed. |
| 32 | Nr. LN ass. | Integer | Number of lymph nodes assessed |
| 33 | Nr. LN aff. | Integer | Number of affected lymph nodes. |
| 34 | Nr. SLND ass. | Integer | Number of assessed sentinel lymph nodes. |
| 35 | Nr. SLND aff. | Integer | Number of affected sentinel lymph nodes. |
| 36 | Histo Nr. | String | Histological sample identification number. |
| 37 | Morph. Code | String | Morphological code of the tumor. |
| 38 | Path. Find. Dt. | Date | Date of pathological staging. |
| 39 | T(p) | String | Pathological tumor classification (TNM: Tumor). |
| 40 | N(p) | String | Pathological nodal classification (TNM: Nodes). |
| 41 | M(p) | String | Pathological metastasis classification (TNM: Metastases) |
| 42 | Stage(p) | String | Overall pathological staging of the tumor. |
| 43 | Thickness | String | Tumor thickness measurement in mm. |
| 44 | Therapy Goal | String | Goal of the therapy. |
| 45 | Therapy Type | String | Type of therapy administered. |
| 46 | Line | Integer | Line of therapy received (sequence of treatments). |
| 47 | Protocol | String | Specific therapy protocol applied. |
| 48 | Sys. Thp. Start | Date | Start date of systemic therapy. |
| 49 | Sys. Thp. End | Date | End date of systemic therapy. |
| 50 | Sys. Thp. End Rsn. | String | Reason for ending systemic therapy. |
| 51 | Progress | String | Patient's tumor response to therapy based on clinical assessment. |
| 52 | Progress Date | Date | Year in which tumor response assessment was conducted |
| 53 | Biobank | String (URL) | Link to biobank consent information. |
| 54 | ADO-Reg/Daten | String (URL) | Link to registry consent data. |

**Table S1: Overview of all dashboard table columns:** This table provides an overview of all columns displayed in the Grafana dashboard, including their names, data types, and descriptions. The table is structured to present key clinical and pathological information about melanoma patients, such as tumor characteristics, mutation status, therapy details, and disease progression.

# Table S2: Overview of all dashboard filtering variables

|  | **Variable Name** | **Values** | **Description** |
| --- | --- | --- | --- |
| 1 | deidentify | yes, no | Filters whether de-identified data is displayed. |
| 2 | tumor_id | - | Selects specific tumor cases by unique identifier. |
| 3 | deceased | All, true, false | Filters patients based on their survival status. |
| 4 | prim_case | All, yes, no | Includes only primary or non-primary cases. |
| 5 | prim_year | 1990 to 2025 | Filters patients by the year of primary tumor diagnosis. |
| 6 | stage | III, IV | Displays patients with selected clinical tumor stages. |
| 7 | primary_location | All, Akrale Lokalisation, Auge, Haut, okkult, Schleimhaut | Filters patients based on the primary tumor location. |
| 8 | primary_location_detail | All, Abdomen, Anus, äußeres Gesäß, Bindehaut, Brust/Oberbauch, Finger/Zehen, Flanke, Fuß, Gesäß, Gesicht, Hals, Hand, Hand-/Fußrücken, Mund, Nase, Oberarm (inkl. Ellenbogen), Oberschenkel (inkl. Knie), Palmar/plantar, Rücken, Schulter, Sonstige, Sonstiger Kopf, subungual, Unbekannt, unbekannt, Unterarm, Unterschenkel, Vagina, Vulva | Filters by specific anatomical tumor location. |
| 9 | histology | All, Akrolentiginöses Melanom, Bindehaut MM, Lentigo Maligna Melanom, Melanom, Melanoma in situ, Melanom auf kongenitalem Nävus, Noduläres Melanom, Sonstige, Superfiziell spreitendes Melanom | Selects patients based on tumor histology type. |
| 10 | ulceration | All, true, false | Filters patients by the presence of tumor ulceration. |
| 11 | braf | 466V, D201H, D201N, D594N, E586K, F242S, G366E, G466A, G466E, G469A, G469E, G469R, G469V, G73R, G76A, G76E, G76V, H181Y, K208E, K601E, K601N, L204S, L584F, L597R, L597S, M91I, N188S, N93_P97del8, Not Determined, P141L, P27S, P74L, R178*, R308K, R44Q, S214F, S363F, S365L, S467L, S616F, S74L, T599_V600insT, V246I, V600, V600D, V600E, V600G, V600K, V600R, V600_K601>E, V600_K601delins, Wildtype | Filters patients by specific BRAF mutation status. |
| 12 | nras | F156L, G12A, G12C, G12D, G12S, G12V, G13D, G13R, Mutated, Nachweis, Not Determined, P110S, P140S, p.Gln61lys, Q61H, Q61K, Q61L, Q61R, Wildtype | Filters patients by specific NRAS mutation status. |
| 13 | kit | L783V, N655S, N822H, N822K, Not Determined, P363L, Q575_L576delins, R634Q, R791I, R888Q, R956Q, S476C, S480A, T67S, V399I, V50L, V530I, V559A, V559D, W582*, Wildtype | Filters patients by specific KIT mutation status. |
| 14 | op_type | All, Fernmetastasen, In transit-Metastasen, Lokalrezidiv, Lymphknotenmetastasen, Nachexzision, Primärtumor, Satellitenmetastasen, Sentinel | Displays patients based on the type of surgery performed. |
| 15 | op_year | 1984 to 2025 | Filters patients by the year of surgery. |
| 16 | therapy_protocol | E.g., Adalimumab, Bevacizumab, Binimetinib, Certolizumab, Dabrafenib, Dabrafenib/Trametinib, Encorafenib/Binimetinib, Imatinib, Ipilimumab, Lenvatinib/Pembrolizumab, …, Nivolumab | Selects patients treated with specific systemic therapies. |
| 17 | therapy_line | 1, 2, 3, 4, 5, 6, 7, 8 | Filters patients based on their systemic therapy line. |
| 18 | sys_start_thpy_year | 1997 to 2025 | Filters patients by the start year of systemic therapy. |
| 19 | therapy_type | All, Antikörper-/Immuntherapie, Chemo- + Immun-/Antikörpertherapie, Chemotherapie, Zielgerichtete Substanzen | Selects patients by the type of therapy received. |
| 20 | therapy_time | Adjunvanz  Adjuvanz  Nicht-Adjunvanz  Nicht-Adjuvanz | Filters patients by whether therapy was adjuvant or not. |
| 21 | tumor_response | B = Klinische Besserung des Zustandes, Kriterien für Teilremission jedoch nicht erfüllt (minimal Response, MR)  B = klinische Besserung des Zustandes, Teilremissionkriterien jedoch nicht erfüllt (minimal response, MR)  D = divergentes Geschehen  E = Entfällt, da Behandlung im Rahmen eines multimodalen Konzepts und dieses noch nicht abgeschlossen  F = Tumorfrei (Nach Resektion)  K = keine Änderung (no change, NC) = stable disease  P = Progression (Fortschreiten der Erkrankung)  P = Progression (Tumorwachstum > 25% und/oder Fortschritt der Tumorerkrankung)  R = Vollremission mit residualen Auffälligkeiten (CRr)  T = Teilremission (partial remission, PR)  U = Beurteilung unmöglich  V = Vollremission (complete remission, CR)  V = Vollremission (Verschwinden des Tumors/Resttumors durch Radiatio und/oder Chemotherapie)  X = Fehlende Angabe  Y = Rezidiv (jedes Wiederauftreten der Erkrankung bei vorheriger kompletter klinischer Tumorfreiheit (biochemisches Rezidiv, Lokalrezidiv und/oder Metastasierung) | Displays patients based on their tumor response to therapy. |
| 22 | tumor_response_year | E.g., 2012, 2013, …, 2022, 2023 | Filters patients by the year of tumor response assessment. |
| 23 | morph_code | M-8000/0 to M-9989/3 | Filters patients by tumor morphology classification. |
| 24 | clin_stage_c | /, 0, 1A, IA, IB, IIA, IIb, IIB, IIC, III, IIIA, IIIB, IIIC, IIID, IV, IVa, IVA, IVB, IVC | Filters patients by clinical stage before treatment. |
| 25 | clinical_t | /, 0, 1, 1a, 1b, 2, 2a, 2b, 2c, 3, 3a, 3b, 4, 4a, 4b, x, X | Selects patients based on tumor size and extent (TNM). |
| 26 | clinical_n | /, 0, 1, 1a, 1b, 1c, 2, 2a, 2b, 2c, 3, 3a, 3b, 3c, x, X | Filters patients by lymph node involvement (TNM). |
| 27 | clinical_m | /, 0, 1, 1a, 1b, 1c, 1d, x | Filters patients by presence of distant metastases (TNM). |
| 28 | clin_stage_p | /, 0, 1A, I, IA, IB, II, IIA, IIB, IIC, III, IIIA, IIIB, IIIC, IIID, IV, IVA, IVB | Filters patients by pathological stage after surgery. |
| 29 | at_first_diagnosis | All, true, false | Includes patients based on data from initial diagnosis. |

**Table S2: Overview of all dashboard filtering variables**: This table outlines the filtering variables available in the Grafana dashboard, including their names, possible values, and descriptions. These filters allow users to refine the displayed patient cohort based on specific criteria such as tumor stage, genetic mutations, therapy type, and clinical outcomes.
